# Cryo-EM structure of apo-form human DNA polymerase δ elucidates its minimal DNA synthesis activity without PCNA

**DOI:** 10.1016/j.jbc.2025.108342

**Published:** 2025-02-22

**Authors:** Yeonoh Shin, Mark Hedglin, Katsuhiko S. Murakami

**Affiliations:** 1 Department of Biochemistry and Molecular Biology, The Center for RNA Molecular Biology, The Center for Structural Biology, The Pennsylvania State University, University Park, Pennsylvania, USA; 2 Department of Chemistry, The Center for Structural Biology, The Pennsylvania State University, University Park, Pennsylvania, USA

**Keywords:** cryo-electron microscopy, DNA polymerase, DNA replication, PCNA, structural biology

## Abstract

DNA polymerase **δ** (Pol **δ**) is a key enzyme in eukaryotic DNA replication and genome maintenance, essential for lagging strand synthesis, leading strand initiation, and DNA repair. While human Pol **δ** exhibits high activity and processivity in its holoenzyme form complexed with proliferating cell nuclear antigen (PCNA), it shows minimal DNA synthesis activity without PCNA, the molecular basis of which remains unclear. Here, we present the cryo-EM structure of the apo-form human Pol **δ**, comprising the catalytic subunit p125 and regulatory subunits p66, p50, and p12, at an overall resolution of 3.65 Å. We identified an acidic α-helix at the N terminus of p125, which occupies the single-stranded DNA-binding cavity within the polymerase domain in the apo-form Pol **δ**. This interaction likely inhibits DNA binding in the absence of PCNA, explaining the low activity of apo-form Pol **δ**. The acidic α-helix is absent in yeast Pol **δ**, providing a molecular explanation for species-specific differences in PCNA-independent Pol **δ** activity. These findings provide critical insights into the regulatory mechanisms of Pol **δ** and its reliance on PCNA for efficient DNA synthesis.

DNA replication and repair in eukaryotes require the coordinated action of 15 distinct DNA polymerases to ensure genome stability and fidelity (1). Among these, DNA polymerase δ (Pol δ) plays an essential role in DNA synthesis during lagging strand DNA replication, the initiation and termination of leading strand replication, and various DNA repair pathways (1, 2, 3, 4). Pol δ is a member of the B-family DNA polymerases and exhibits activity, fidelity, and processivity in the replication fork complex, making it indispensable for genome maintenance. Pol δ functions as a heteromultimeric complex (2, 5). In baker’s yeast (*Saccharomyces cerevisiae*), Pol δ is comprised of a catalytic subunit (Pol 3) and two regulatory subunits (Pol 31 and Pol 32). In humans, Pol δ is comprised of a catalytic subunit (POLD1/p125) and three regulatory subunits (POLD2/p50, POLD3/p66, and POLD4/p12). Pol δ forms a holoenzyme complex with proliferating cell nuclear antigen (PCNA) that is critical for efficient DNA synthesis. PCNA functions as a sliding clamp, increasing the processivity of Pol δ by tethering it to DNA (6, 7, 8).

While the importance of PCNA for Pol δ activity is well established, significant differences in PCNA dependency exist across species. *S*. *cerevisiae* Pol δ retains measurable DNA synthesis activity in the absence of PCNA (8), whereas human Pol δ is nearly inactive under similar conditions (9, 10, 11). This discrepancy suggests fundamental differences in the structural and mechanistic regulation of Pol δ across species. Despite previous structural studies of the human Pol δ in complex with PCNA and primer/template DNA (p/t DNA) (6), the molecular basis for its minimal activity in the apo form remains unclear. To address this question, we determined the cryo-EM structure of the apo-form human Pol δ, consisting of p125, p66, p50, and p12 subunits. Our study reveals a novel structural feature: an acidic α-helix at the N terminus of p125 that occupies the DNA-binding cavity of its polymerase domain. This interaction likely inhibits DNA binding to human Pol δ in the absence of PCNA, providing a mechanistic explanation for the low activity of apo-form human Pol δ. Importantly, this feature is absent in *S. cerevisiae* Pol δ, offering insights into species-specific differences in Pol δ activity. These findings provide a foundation for future investigations into Pol δ function and its regulation in diverse biological systems.

## Results

### Expression and purification of human Pol **δ**

**Figure 1:**
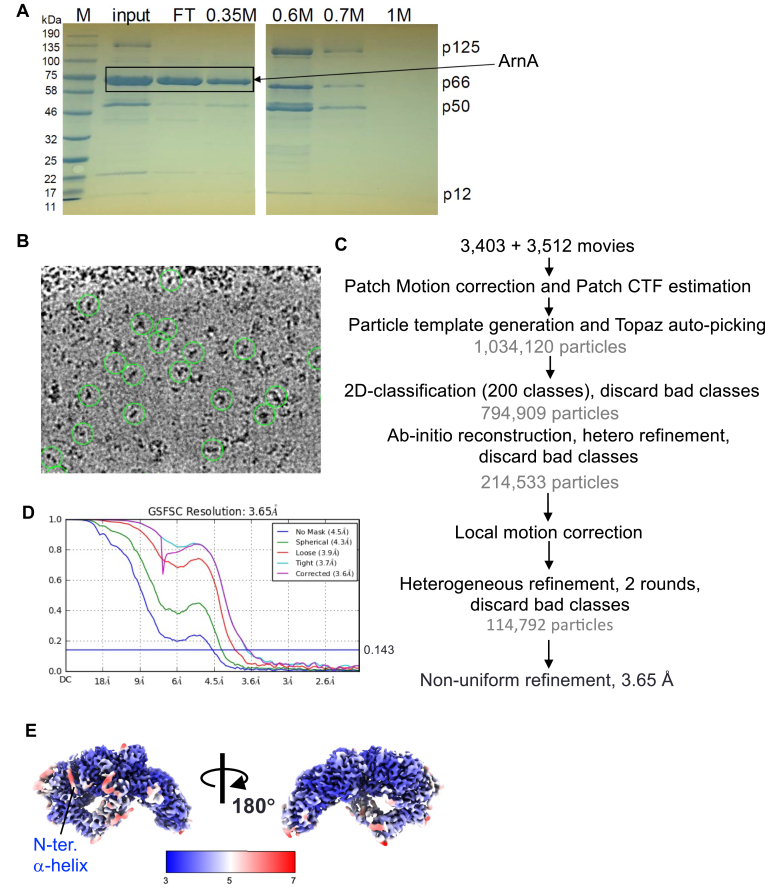
**Cryo-EM structure determination of the apo-form human Pol δ.***A*, SDS-PAGE gel analysis of human Pol δ purification. Input, flow-through (FT), and elution fractions from heparin column chromatography are shown. Subunits of Pol δ and ArnA, a contaminant from *Escherichia coli*, are labeled. *B*, representative micrograph of human Pol δ. Particles used for reconstruction of the cryo-EM map are indicated by *green circles*. *C*, cryo-EM data processing flowchart, illustrating motion and CTF correction, particle picking, 2D classification, *ab initio* reconstruction, heterogenous and nonuniform refinement steps. *D*, Fourier shell correlation (FSC) plot with a 0.143 FSC criterion, indicating the estimated resolution of the map. *E*, local resolution map of Pol δ, highlighting the N-terminal α-helix of the p125 subunit. CTF, contrast transfer function; Pol δ, DNA polymerase δ.

Recombinant human Pol δ was successfully expressed in *Escherichia coli* cells (10) in the presence of ferric ammonium citrate and cysteine as supplements for efficient incorporation of an iron–sulfur (Fe–S) cluster in the p125 subunit and purified as a heterotetramer consisting of p125, p66, p50, and p12 subunits. The protein was purified using a combination of Ni-affinity, Q-sepharose, size-exclusion, and heparin column chromatography. Removal of ArnA, a common *E. coli* contaminant after Ni-affinity chromatography (12), was achieved during the heparin chromatography step (Fig. 1*A*). The purified complex was analyzed *via* SDS-PAGE, confirming the presence of all four Pol δ subunits.

### Cryo-EM structure of the apo-form human Pol **δ**

**Figure 2:**
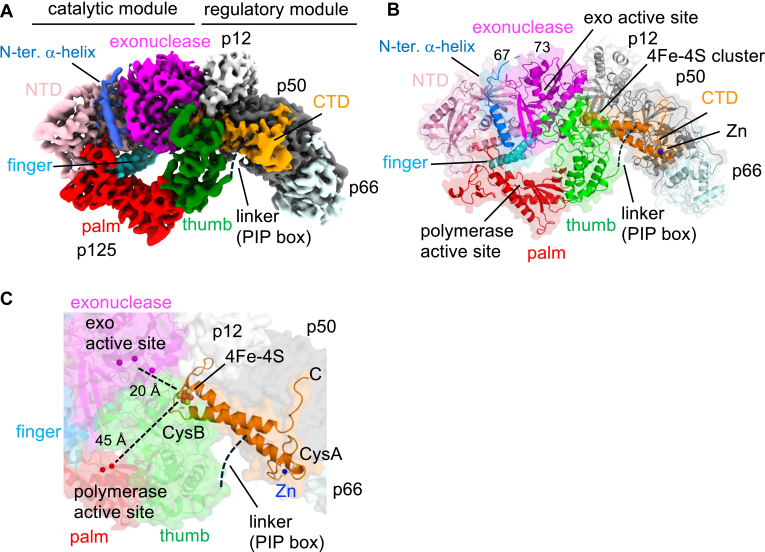
**Cryo-EM structure of the apo-form human Pol δ.***A*, the cryo-EM image and (*B*) the 3D structure (*cartoon model* with transparent surface) of Pol δ showing its subunit organization (p125, p66, p50, and p12), modules (catalytic and regulatory), domains (NTD, exonuclease, polymerase, and CTD), and subdomains (thumb, finger, and palm) within the polymerase domain of p125 subunit. *C*, close-up view of the p125-CTD. Locations of the polymerase and exonuclease active sites are labeled (Mg^2+^ ions coordinated at the polymerase and exo active sites are shown as *red* and *magenta spheres*, respectively), and distances from the 4Fe–4S cluster are indicated. Zn^2+^ (*blue sphere*) and 4Fe–4S cluster (*orange and yellow spheres*) coordinated by Cys clusters (CysA and CysB) are indicated. A disordered PIP-box linker region is indicated. CTD, C-terminal domain; Fe–S, iron–sulfur; NTD, N-terminal domain; PIP, proliferating cell nuclear antigen–interacting peptide; Pol δ, DNA polymerase δ.

The cryo-EM structure of human Pol δ was determined at an overall resolution of 3.65 Å (Fig. 1, *B*–*E*). The structure revealed a fan-shaped architecture (∼160 × 100 × 80 Å) comprising the catalytic module (p125 residues 1–993) and the regulatory module, which includes the carboxyl-terminal domain (CTD) of p125 (residues 1007–1107) and the three regulatory subunits (p66, p50, and p12) (Fig. 2*A*, Movie S1). The catalytic module is further divided into the amino-terminal domain (N-terminal domain, residues 1–310, 528–570), exonuclease domain (residues 311–527), and polymerase domain (residues 571–990), which includes thumb (residues 829–990), palm (residues 571–653, 706–828), and finger subdomains (residues 654–705) (Fig. 2*B*, Movie S2).

Notably, the cryo-EM density of the linker region containing the PCNA-interacting peptide (PIP-box) in the p125 subunit was disordered, suggesting flexibility prior to binding PCNA (6, 13). The p125-CTD plays a critical role in maintaining the integrity of the complex, forming extensive interactions with the catalytic module and the regulatory subunits (Fig. 2*B*). The p125-CTD coordinates Zn^2+^ by CysA cluster (residues 1012, 1015, 1026, and 1029) and 4Fe–4S cluster by CysB cluster (residues 1058, 1061, 1071, and 1076) (Fig. 2*C*). 4Fe–4S clusters provide redox potential for several enzymatic reactions; however, the 4Fe–4S cluster in Pol δ is located 20 and 45 Å away from its exonuclease and polymerase catalytic centers, respectively, rendering it unlikely to be involved in any catalytic activity of Pol δ. Loss of the 4Fe–4S cluster binding motif of Pol δ reduces its polymerase activity (14, 15), suggesting a structural role in the Pol δ assembly and/or folding as observed in other nucleic acid enzymes (16), and potentially sensing the intracellular oxidation–reduction state.

### Structural features of the N-terminal region of p125 explain species-specific differences in Pol **δ** activity

**Figure 3:**
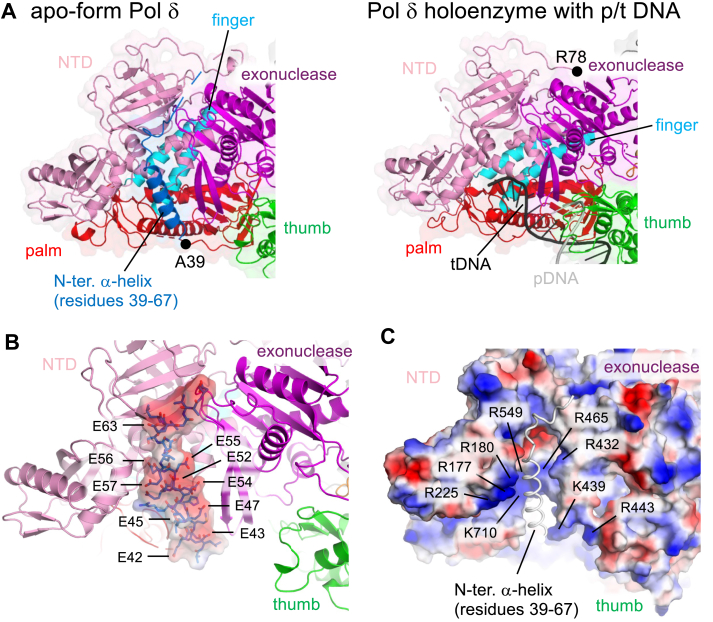
**Structural role of the N-terminal α-helix in human Pol δ.***A*, comparison of the template DNA-binding cavity in the apo-form (*left*) and holoenzyme (*right*, PDB code: 6TNY). The N-terminal α-helix in the apo-form is labeled. Positions of the N termini of p125 subunit in the apo-form (*left*, A39) and the holoenzyme (*right*, R78) are indicated by *black circles*. *B*, electrostatic surface of the N-terminal α-helix of p125 accommodated in the template DNA-binding channel of human Pol δ (*blue*: negative; *red*: positive). Positions of acidic residues are indicated. *C*, electrostatic surface of the template DNA-binding channel of human Pol δ (*blue*: negative; *red*: positive). Basic residues on the surface are labeled. The orientation of this panel is the same as in (*B*). PDB, Protein Data Bank; Pol δ, DNA polymerase δ.

**Figure 4:**
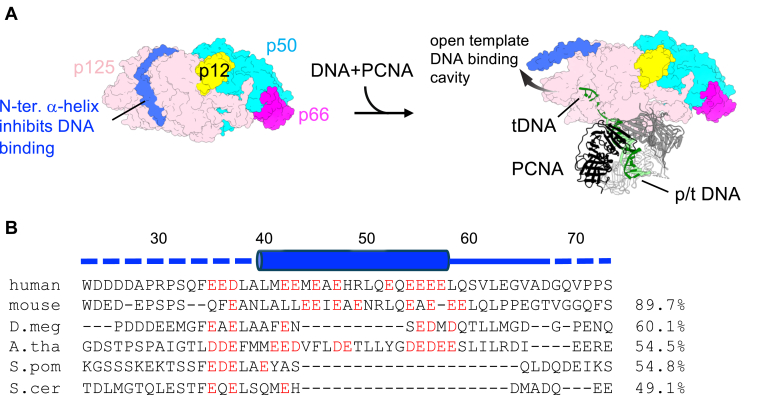
**Structural basis of minimal DNA synthesis activity of human Pol without PCNA.***A*, model illustrating how the N-terminal α-helix inhibits the productive DNA complex formation of human Pol δ in the absence of PCNA. *B*, sequence alignment of the N-terminal regions of large subunit of Pol δ from human (NP_001243778.1), mouse (NP_035261.3), *Drosophila melanogaster* (D. meg: NP_524099.2), *Arabidopsis thaliana* (A. tha: NP_201201.2), *Schizosaccharomyces pombe* (S. pom: NP_596124.1), and *Saccharomyces cerevisiae* (S. cer: AJU99726.1). Acidic residues around the N-terminal α-helix (depicted as a *cylinder*) are colored *red*. The sequence identity (%) of the largest subunit of full-length Pol δ relative to human Pol δ is indicated on the *right*. PCNA, proliferating cell nuclear antigen; Pol δ, DNA polymerase δ.

A striking feature observed in the apo-form human Pol δ was the presence of an acidic α-helix at the N terminus of p125 (residues 39–58). This α-helix occupies the single-stranded DNA-binding cavity of the polymerase domain of Pol δ (Fig. 3*A*, *left*). Most residues forming the α-helix are acidic, allowing electrostatic interactions with the positively charged DNA-binding cavity (Fig. 3, *B* and *C*). Local resolution of the α-helix is relatively low (Fig. 1*E*) suggesting its weak binding to the DNA-binding cavity of the polymerase domain. The N-terminal α-helix is absent in the cryo-EM structures of human Pol δ holoenzymes bound to p/t DNA, where this region is disordered until residue R78 (Fig. 3*A*, *right*) (6). Sequence alignment of the N-terminal region of p125 in human Pol δ and Pol3 in yeast Pol δ revealed that the acidic α-helix is absent in yeast (Fig. 4*B*). This observation aligns with experimental data showing that yeast Pol δ retains DNA synthesis activity in the absence of PCNA (8), whereas human Pol δ is nearly inactive under similar conditions (9, 10).

To further test the hypothesis that the N-terminal tail of the p125 subunit inhibits the DNA-binding activity of human Pol δ, we attempted to prepare a deletion mutant lacking the first 70 amino acids of the p125 subunit. This mutant was expressed using the same *E. coli* system that successfully produced wildtype Pol δ. However, the mutant exhibited significantly reduced expression levels, and *E. coli* cells transformed with the mutant expression vector displayed very poor growth. These observations suggest that it might engage in nonspecific interactions with the *E. coli* genome and/or expression vector, leading to cytotoxic effects and compromised expression. This aligns with the structural findings of this study, which indicate that the N-terminal tail of p125 prevents nonspecific DNA interactions by occupying the DNA-binding cavity in the apo-form Pol δ. We are currently exploring alternative expression systems to produce this mutant and will report the results in a separate publication in the future.

## Discussion

The cryo-EM structure of apo-form human Pol δ (Fig. 2) provides critical insights into its minimal DNA synthesis activity in the absence of PCNA. A key discovery is the acidic α-helix at the N terminus of p125, which occupies the single-stranded DNA-binding cavity in the polymerase domain (Fig. 3). This interaction directly competes with DNA binding to the apo-form Pol δ, thus minimizes its activity, requiring PCNA-mediated anchoring to promote productive interactions with DNA (Fig. 4*A*). The flexibility of the PIP-box in p125 subunit and its disordered state in the apo-form suggest a structural rearrangement upon binding to PCNA encircling, which may facilitate the displacement of the N-terminal α-helix from the DNA-binding cavity. This mechanism could explain how PCNA enhances both the activity and processivity of human Pol δ. The absence of the N-terminal acidic α-helix in yeast Pol δ provides a molecular basis for the species-specific differences in PCNA-independent Pol δ activity. Yeast Pol δ likely maintains the DNA-binding site of the polymerase domain even without PCNA, whereas human Pol δ relies on PCNA to displace the inhibitory α-helix and establish productive DNA interactions.

This study not only resolves a longstanding question regarding the poor activity of apo-form human Pol δ but also highlights the role of the N-terminal region of p125 in regulating DNA binding. The amino acid sequence of basic α-helix is conserved in mammal (mouse) and plant (*Arabidopsis thaliana*) but not in insect (*Drosophila*) or fission yeast (Fig. 4*B*), suggesting that the poor activity of Pol δ in the absence of PCNA is a characteristic specific to higher eukaryotes. Future studies will aim to experimentally test the functional and activity impacts of the N-terminal α-helix of human Pol δ *in vitro* and *in vivo*.

## Experimental procedures

### Expression and purification of human Pol **δ**

BL21-codonplus (DE3)-RP cell (Stratagene) was cotransformed with pET-POLD4/1 and pGBM-POLD2/3 for expressing all four subunits of the human Pol δ (10). Although none of subunit has His-tag, human Pol δ has an innate affinity to Ni-column due to p66 subunit binding for this resin. Seed culture (500 ml of Terrific broth media) was prepared with 250 μg/ml ampicillin, 20 μg/ml streptomycin, and 30 μg/ml chloramphenicol at 37 °C until an absorbance of ∼1 at 600 nm, which was transformed to the Sartorius Biostat D-DCU 100 L vessel (Penn State Huck CSL Behring Fermentation Facility) containing Terrific broth media with antibiotics at 37 °C. Cells were grown until an absorbance of ∼1 at 600 nm, and then the growth temperature was reduced to 18 °C followed by adding ferric ammonium citrate and cysteine each to a final concentration of 0.5 mM as supplements for the 4Fe–4S cluster incorporation to p125 subunit. Proteins were expressed by adding 0.2 mM IPTG and incubating at 18 °C for 15 h. Hundred liters of fermentation yielded 600 g of cell paste.

Hundred grams of cell paste were resuspended in a 230 ml lysis buffer (50 mM Hepes–NaOH, pH 7.5, 500 mM NaCl, 10 mM beta-mercaptoethanol, 0.1 mM EDTA, 1 mM PMSF, 1 Roche complete EDTA-free protease inhibitor tablet) and lysed by sonication. The lysate was clarified by spinning at 15,000 rpm for 15 min at 4 °C. Imidazole was added to a final concentration of 5 mM to the supernatant followed by adding prechilled 10% (w/v) streptomycin sulfate dropwise to final 1% while stirring at 4 °C for 30 min. After spinning the solution at 20,000 rpm for 30 min, the supernatant was loaded to a 5 ml HiTrap-column (GE Healthcare) equilibrated in a buffer N (50 mM Hepes–NaOH, pH 7.5, 500 mM NaCl, 10% glycerol, 10 mM beta-mercaptoethanol) plus 5 mM imidazole followed by elution with the buffer N plus 100 mM imidazole. NaCl concentration of the eluted fraction was diluted to 100 mM and loaded to a 5 ml Q-HP column (GE Healthcare) equilibrated with Buffer QH (50 mM Hepes–NaOH, pH 7.5, 100 mM NaCl, 10% glycerol, 10 mM beta-mercaptoethanol). The protein was eluted over 100 to 800 mM NaCl gradient. The fractions containing Pol δ were pooled and loaded onto a 120 ml Superdex200 (16/600) size-exclusion column (GE Healthcare) equilibrated with buffer S (50 mM Hepes–NaOH, pH 7.5, 400 mM NaCl, 10% glycerol, and 1 mM DTT). The fractions containing Pol δ were pooled, diluted to 100 mM NaCl, and loaded into a 1 ml HiTrap Heparin column (GE Healthcare) equilibrated with Buffer QH. Pol δ was eluted with Buffer QH plus 600 mM NaCl. The sample was concentrated to 7 mg/ml and stored at −80 °C until preparing cryo-EM grid.

### Cryo-EM grid preparation and data acquisition

Immediately before preparing cryo-EM grid for screening and data collection, the buffer was exchanged to 10 mM Hepes–NaOH (pH 7.5), 120 mM NaCl, and 2% glycerol followed by the addition of 8 mM of CHAPSO to the 6 μM of Pol δ. A 3.5 μl of the sample was applied to a glow-discharged C-Flat Holey Carbon grid (CF-2/1-4Cu-50), blotted, and plunge-frozen in liquid ethane using a Vitrobot Mark IV (FEI) with 100% humidity at 4 °C. The cryo-EM data were collected using a 300 keV Titan Krios (Thermo Fisher) microscope equipped with a K3 direct electron detector (Gatan) at the National Cancer Institute Cryo-EM Facility at Frederick. The defocus range was −1.0 to −2.5 μm, and the magnification was 81,000x in electron counting mode (pixel size = 1.12 Å/pixel). Forty frames per movie were collected with a nominal dose of 50 e^−^/Å^2^.

### Cryo-EM data processing

The data were processed using cryoSPARC (Fig. 1*C*, Table 1) (17). The movies were aligned and dose-weighted using Patch-motion correction and Patch-contrast transfer function estimation (18, 19) followed by discarding low-quality micrographs that had large motions or poor contrast transfer function resolution through manual curate exposure job. A structure of apo-form human Pol δ was obtained from a structure of human Pol δ–PCNA–p/t DNA (Protein Data Bank [PDB] code: 6TNY) (6) by removing PCNA and DNA and converted to an electron density map using Eman2. The map was imported to the cryoSPARC, and 50 2D class templates were generated for autotemplate picking. About 1,705,644 particles were picked and extracted with a box size of 320 and subjected to 2D classification followed by multiple rounds of *ab initio* and heterogenous refinements to remove poorly populated classes. A class representing Pol δ (81,653 particles) was selected and refined to generate a homogenous density map at 4.16 Å resolution. To improve particle picking, Topaz (20) was utilized with the particles used. About 1,034,120 particles were re-extracted from the movies and subjected to 2D classification to 200 classes. Ab initio was performed with 62 poorly populated 2D classes (794,909 particles) to generate six poorly populated seed volumes followed by heterogenous refinement with the map1 and six *ab initio* volumes as reference models. The class corresponding to Pol δ was selected and refined to a 3.88 Å density map (214,533 particles) (Fig. 1*C*). To validate and polish the map, two more rounds of *ab initio* to two classes were performed without providing references. The class corresponding to Pol δ (114,792 particles) was selected and refined. The nominal resolution of the cryo-EM map was estimated by 0.143 gold standard Fourier shell correlation cutoff (Fig. 1*D*).

**Table 1:** Summary for cryo-EM data collection and 3D structure refinement

	Human Pol δ (Electron Microscopy DataBank code: EMD-48117) (PDB code: 9EKB)
Data collection and processing	
Magnification	81,000
Voltage (kV)	300
Electron exposure (e^−^/Å^2^)	50
Defocus range (μm)	−1.0 to −2.5
Pixel size (Å)	1.12
Symmetry imposed	C1
Initial particle images (no.)	1,034,120
Final particle images (no.)	114,792
Map resolution (Å)	3.65
FSC threshold	0.143
Map resolution range (Å)	3.2–7.8
Refinement	
Initial model used (PDB code)	6TNY
Model resolution (Å)	3.65
FSC threshold	0.143
Map sharpening B-factor (Å^2^)	−92.5
Model composition	
Nonhydrogen atoms	13,325
Protein residues	1694
Ligands	Zn: 1, 4Fe–4S: 1
B-factors (Å^2^) (mean)	
Protein	142.15
Ligand	147.76
R.m.s. deviations	
Bond lengths (Å)	0.003
Bond angles (°)	0.468
Validation	
MolProbity score	1.7
Clash score	4.73
Rotamer outliers (%)	1.91
Ramachandran plot	
Favored (%)	96.36
Allowed (%)	3.52
Disallowed (%)	0.12
Q score	
All	0.4320
Chain A (p125)	0.4160
Chain B (p50)	0.4680
Chain C (p66)	0.4260
Chain D (p12)	0.4570

### Model building and structure refinement

Initial model was built by superposing the human Pol δ holoenzyme (PDB code: 6TNY) (6) on the cryo-EM density map. Missing amino acid residues were manually added, and modeling errors found in the human Pol δ holoenzyme were corrected by using Coot (21). The structure was real-space refined by using rigid-body refinement, secondary structure, Ramachandran, and rotamer in Phenix (22).

## Data availability

The cryo-EM density maps and the refined model have been deposited in the Electron Microscopy DataBank (www.ebi.ac.uk/emdb/) and PDB (www.rcsb.org) under accession numbers EMD-48117 and 9EKB, respectively.

## Supporting information

This article contains supporting Information.

## Conflict of interest

The authors declare that they have no conflicts of interest with the contents of this article.
