# Supplementary material for: Cryo-EM structure of apo-form human DNA polymerase δ elucidates its minimal DNA synthesis activity without PCNA
Source: J Biol Chem. 2025 Feb 22;301(4):108342. doi: 10.1016/j.jbc.2025.108342 (PMC11982976; doi:10.1016/j.jbc.2025.108342)
Supplement: Supporting information [file mmc1.docx]

**SUPPLEMENTAL INFORMATION**

SMovie 1. Cryo-EM density map of the apo-form human Pol δ. Subunit composition. Related to Fig. 2a.

SMovie 2. Cryo-EM density map of the apo-form human Pol δ. Domain and sub-domain organization. Related to Fig. 2b.
